# Clinical outcome of cerebrospinal fluid shunts in patients with leptomeningeal carcinomatosis

**DOI:** 10.1186/s12957-019-1595-7

**Published:** 2019-03-27

**Authors:** Hye Seon Kim, Jong Bae Park, Ho-Shin Gwak, Ji-Woong Kwon, Sang-Hoon Shin, Heon Yoo

**Affiliations:** 1 Department of Neurosurgery, Seoul National University College of Medicine, Seoul, Republic of Korea; 2 Department of Biomedical Science, National Cancer Center, Graduate School of Cancer Science and Policy, National Cancer Center, Goyang, Republic of Korea; 3 Department of Cancer Control, National Cancer Center, Graduate School of Cancer Science and Policy, National Cancer Center, 323 Ilsan-ro, Ilsandong-gu, Goyang-si, Gyeonggi-do 10408 Republic of Korea; 4 Neuro-oncology Clinic, National Cancer Center, Goyang, Republic of Korea

**Keywords:** Cerebrospinal fluid, Complication, Efficacy, Leptomeningeal carcinomatosis, Shunt

## Abstract

**Background:**

Leptomeningeal carcinomatosis (LMC) is frequently associated with hydrocephalus, which quickly devastates the performance of the patient. Cerebrospinal fluid (CSF) shunt is a widely accepted treatment of choice, but the clinical outcomes in patients with LMC are not well studied. This study aimed to examine the efficacy of a CSF shunt in patients with LMC.

**Methods:**

Seventy patients with LMC confirmed by cytology or magnetic resonance imaging (MRI) underwent ventriculoperitoneal (VP) or lumboperitoneal (LP) shunt surgery. We retrospectively analyzed the clinical characteristics of patients, symptom improvement after the shunt, rate of complications associated with the surgery, and overall survival.

**Results:**

Fifty-five patients had systemic cancer as a preceding disease, including lung cancer (45), breast cancer (6), and others (4). Primary brain tumors were mainly glioma (7) and medulloblastoma (5). Fifty-one patients had VP shunt, and 19 had LP shunt. After surgery, preoperative symptoms “improved” in 35 patients (50%) and were “normalized” in 24 of those patients (34%). Shunt malfunction occurred in eight patients, and infection occurred in eight patients. Seventeen patients underwent revision due to infection, shunt malfunction, or over-drainage. There were no complications associated with peritoneal seeding during a median follow-up of 3.3 months after surgery. The median overall survival was 8.7 months (95% confidence interval, 6.0–11.4) from LMC diagnosis and 4.1 months from shunt surgery.

**Conclusion:**

VP or LP shunt is effective for patients with hydrocephalus from LMC in terms of symptom improvement and prolonging of overall survival with an acceptable rate of procedure-related complications.

**Trial registration:**

This study was approved by the Institutional Review Board (IRB) of the National Cancer Center (retrospectively registered, NCC2018-0051).

## Background

Leptomeningeal carcinomatosis (LMC), a terminal disease defined by spreading of cancer cells in the cerebrospinal fluid (CSF), affects 5–10% of patients with solid tumors, and the most notorious of which are melanoma; breast, lung, and ovarian cancers; and primary brain tumors [1].

LMC has a poor prognosis as the life expectancy after diagnosis is 4–6 weeks without treatment [1, 2]. LMC is challenging to treat in many ways. Radiation to local field has a limited role, as the disease is disseminated along the neuraxis [3]. Systemic chemotherapy is ineffective because of the blood-brain barrier [4]. Intra-CSF chemotherapy, administered either intrathecally or intraventricularly, can maintain therapeutic drug concentrations in the CSF for 48–72 h [5]; however, a marginal survival benefit of 3–6 months and suspicious neurotoxicity raise questions about its effectiveness [6–8].

Infiltration of the CSF space by cancer cells may obstruct CSF pathways and cause CSF malabsorption, resulting in increased intracranial pressure (ICP) and/or hydrocephalus [9]. The common presenting symptoms of LMC include headache, nausea, vomiting, and altered mentality, all of which are related to increased ICP and hydrocephalus. Increased ICP causes an uneven distribution of intrathecally or intraventricularly administered drugs, making them less effective [10]. Thus, hydrocephalus and increased ICP degrade the performance and prognosis of patients with LMC.

Although CSF shunt surgery is a relatively simple neurosurgical procedure, the percentage of patients who receive it is less than the incidence of increased ICP, and its outcomes are little and rarely reported [11–13]. As the prognoses of patients with LMC are poor, there is a tendency for physicians to decline aggressive treatment such as surgical intervention. Furthermore, complications such as hemorrhage, infection, and device malfunction, along with the risk of peritoneal transfer of cancer cells, are reasons to not perform CSF shunt in patients with LMC [14, 15].

This retrospective study aimed to evaluate the efficacy of CSF shunt in patients with LMC in terms of (1) symptom improvement, (2) malfunction rate, including cancer cell-caused obstruction or spillage, and (3) overall survival (OS) benefit.

## Methods

### Study design

This retrospective review was based on the electronic medical records of 70 consecutive patients that underwent placement of either ventriculoperitoneal (VP) or lumboperitoneal (LP) shunt for treatment of increased ICP or hydrocephalus secondary to LMC between 2002 and 2017 at a single institution, National Cancer Center, Korea. All patients were diagnosed by CSF cytology and had a suggestive or definite finding of LMC [16] on gadolinium-enhanced MRI. This retrospective study was reviewed and approved by the Institutional Review Board of the National Cancer Center of Korea (NCC2018-0043).

#### Clinical parameters and operative procedures

Demographic data, primary tumor type, preoperative Karnofsky performance status (KPS) score, and type of the shunt (VP vs. LP) were analyzed as possible factors affecting the shunt results. Shunt procedures were performed routinely as described in the literature [13]. The operating physicians chose the type of shunt and entry point (in case of VP shunt). The type of reservoir (fixed vs. programmable) was determined based on the availability or ICP.

#### End points

Symptom improvement was evaluated at the time patients were discharged from the hospital after shunt surgery and defined as follows: (1) “normalized,” all hydrocephalus-related symptoms were clearly solved; (2) “improved,” preoperative hydrocephalus-related symptoms were improved but remained to some extent; (3) “not improved,” preoperative hydrocephalus-related symptoms were unchanged. Complications were classified as malfunctions or CSF infections that lead to shunt revision. Causes of malfunctions were confirmed either by a shunt function test or by intraoperative findings.

Survival time was calculated from either the date of LMC diagnosis or the day of shunt placement until death or last follow-up visit.

#### Statistical analysis

Parameters were analyzed using the Statistical Package for Social Sciences (SPSS, version 18, Chicago, IL). Categorical variables were compared between the VP shunt and LP shunt using the chi-square test. A *p <* 0.05 indicated statistical significance. A Kaplan-Meier curve was used to analyze survival rates, and a log-rank test was used to evaluate prognostic factors associated with OS.

## Results

### Patient characteristics

Seventy patients (40 females, 30 males) underwent shunt operation during the study period. All patients had extraventricular drainage or lumbar drainage to control increased intracranial pressure or to relieve headache before the shunt procedure. Disease preceding LMC was systemic cancer in 55 patients and primary brain tumors in 15 patients (Table 1). The systemic cancers were non-small cell lung cancer (NSCLC; *n* = 45), breast cancer (*n* = 6), leukemia (*n* = 2), cholangiocarcinoma (*n* = 1), and malignancy of unknown origin (*n* = 1). The primary brain tumors were high-grade glioma (*n* = 7), medulloblastoma (*n* = 5), primary neuroectodermal tumor (*n* = 2), and atypical teratoid/rhabdoid tumor (*n* = 1).

**Table 1:** Characteristics of patients with leptomeningeal carcinomatosis (LMC) receiving cerebrospinal fluid (CSF) shunt operation

Characteristics	All patients (*n* = 70)	Metastases (*n* = 55)	Primary brain tumors (*n* = 15)	*p* value
Median age (range)	53.0 (1–81)	56.0 (34–81)	18.0 (1–66)	< 0.001
Gender				0.62
Male	30	24	6	
Female	40	31	9	
Shunt type				0.11
VP	51	38	13	
LP	19	17	2	
KPS score				0.099
≥ 70	41	35	6	
< 70	29	20	9	

*Abbreviations*: *KPS* Karnofsky performance status, *LP* lumboperitoneal, *VP* ventriculoperitoneal

The median age of the patients was 53 (range, 1–81) years. Patients with primary brain tumors had lower median age than those with systemic cancers (18.0 vs. 56.0 years, *p* < 0.001). The median preoperative KPS score was 70 (range, 40–90).

### Clinical outcomes after surgery

Fifty-one and 19 patients underwent VP shunt and LP shunt, respectively. There was no predilection for shunt type according to preceding disease. Forty-six patients (92%) with VP shunts and 13 patients (68%) with LP shunts had a programmable valve [that difference was because of late availability of programmable valves for LP shunt (since 2012)].

After shunt surgery, preoperative symptoms were “normalized” in 24 patients (34%), “improved” in 35 patients (50%), and “not improved” in 11 patients (16%). The type of shunt used (VP vs. LP), preceding disease (systemic metastases vs. primary brain tumor), and preoperative KPS score (≥ 70 vs. < 70) did not affect the clinical outcomes.

### Complications related to the shunt procedure

Median follow-up time was 3.3 (range, 0.2–43.0; 95% confidence interval, 3.47–6.93) months after the shunt. Neither peritoneal seeding nor secondary ascites were observed in all patients during the follow-up period. Seventeen patients (24%) underwent revision surgery due to malfunction or infection (eight patients each) or to intolerable over-drainage symptoms (one patient, case 17, Table 2). The average time between initial surgery and revision surgery was 1.1 ± 0.86 months. Six patients required a second revision surgery, and two needed a third revision.

**Table 2:** Description of shunt revision due to malfunction and infection

Case no.	Sex/age	Primary ca.	Shunt type/reservoir	Malfunction or infection	Revision
1	M/34	NSCLC	VP/programmable	*S. aureus* infection presented as malfunction with pus discharge around the distal catheter at 1 month	Shunt removal
2	F/61	Breast ca.	LP/programmable	*S. epidermidis* infection after Ommaya MTX injection	Shunt removal and VP shunt 2 months later
3	F/56	Breast ca.	LP/programmable	*S. epidermidis* infection after Ommaya MTX injection	Shunt removal, lumbar drainage, and re-insertion after infection control
4	F/61	NSCLC	LP/programmable	Wound dehiscence after Ommaya MTX injection resulted in *S. epidermidis* infection	Shunt removal, extraventricular drainage, and re-insertion after infection control
5	F/63	NSCLC	LP/programmable	*K. pneumoniae* infection 4 days after the shunt	Shunt removal and extraventricular drainage
6	F/59	NSCLC	LP/programmable	*C. albicans* infection of abdominal wound	Shunt removal and VP shunt
7	F/18	MBL	LP/programmable	*S. epidermidis* infection after Ommaya MTX injection	Shunt removal and re-insertion after infection control
8	F/59	NSCLC	VP/programmable	*S. aureus* infection 6 months after the shunt	Shunt removal
9	F/29	GBL	VP/programmable	Distal catheter obstruction 3 months after the shunt	Distal catheter externalization
10	F/13	MBL	VP/programmable	Ventricular catheter obstruction (myxoid material only)	Extraventricular drainage and re-insertion
11	M/1	MBL	VP/programmable	Proximal catheter migration on 10 days after the shunt	Shunt revision
12	M74	NSCLC	LP/fixed	Under-drainage without obstruction	Catheter irrigation and VP shunt later
13	F/52	NSCLC	LP/fixed	Distal catheter (pre-peritoneal) malposition	Distal wound revision
14	M/52	NSCLC	LP/fixed	Proximal catheter obstruction	LP shunt removal and VP shunt
15	M/54	NSCLC	LP/fixed	Under-drainage without obstruction	LP shunt removal and ventricular Ommaya
16	M/53	AOG	LP/fixed	Distal catheter leakage with reservoir	LP shunt removal and VP shunt
17	F/8	MBL	VP/fixed	Intolerable over-drainage	Revision with programmable valve

*Abbreviations*: *AOG* anaplastic oligodendroglioma, *GBL* glioblastoma, *LP* lumboperitoneal, *MBL* medulloblastoma, *MTX* methotrexate, *NSCLC* non-small cell lung cancer, *VP* ventriculoperitoneal

Infections leading to revision surgery were due to skin contaminants in six patients, four of which had *Staphylococcus epidermidis* infection and a history of methotrexate intraventricular injection via Ommaya reservoir in the presence of LP shunt (cases 2, 3, 4, and 7). The other two patients had VP shunts and had *Staphylococcus aureus* infection at 1 month and 6 months after the shunt surgery, respectively, without any history of reservoir puncture before the infection (cases 1 and 8). One patient that was bedridden had a *Candida albicans* infection of an abdominal wound 2 weeks after LP shunt installation (case 6). Another patient had a *Klebsiella pneumoniae* infection 4 days after LP shunt installation (case 5). The infected LP shunts were removed and either re-inserted after infection control via the same route (four patients) or changed to a VP shunt (two patients). Two patients with VP shunt infection refused to undergo new shunt insertion.

Shunt malfunction occurred in eight patients. In four patients, shunt function study documented the malfunction site. Two patients with VP shunts and glioblastoma (case 9) and medulloblastoma (case 10) showed distal and ventricular catheter obstruction, respectively. No cancer cell obstruction but only myxoid material was found in the ventricular catheter from the patient with medulloblastoma. Two patients with LP shunts with fixed pressure-valve reservoirs had proximal catheter obstruction (case 14) and distal catheter leakage at the junction with the reservoir (case 16), respectively. In another two patients, X-ray examination revealed mechanical failures due to proximal catheter migration (case 11) and distal catheter malposition into the pre-peritoneal space (case 13), respectively. The remaining two patients had LP shunts with fixed pressure-valve reservoirs. Under-drainage was suspected in those patients because of deterioration before revision, although neither obstruction nor malfunction was found during revision surgery. One patient received a VP replacement shunt (case 12), and the other received an intraventricular Ommaya (case 15).

Revision surgery was more common in patients with LP shunts than in those with VP shunts because of the higher rates of malfunction (5/14 vs. 3/48, *p* = 0.017) and infection (2/49 vs. 6/13, *p* < 0.001) with the LP shunts. Five out of seven LP shunts with fixed pressure valve reservoirs malfunctioned, while all 12 LP shunts with programmable valves did not. A possible reason for the higher incidence of infection in the patients with LP shunts is that four out of 11 patients that received Ommaya intraventricular chemotherapy had *S. epidermidis* infection.

Five patients had medulloblastoma, and none of them showed any sign of peritoneal seeding during 2.9–40.3 months of follow-up.

### Overall survival

**Fig. 1:**
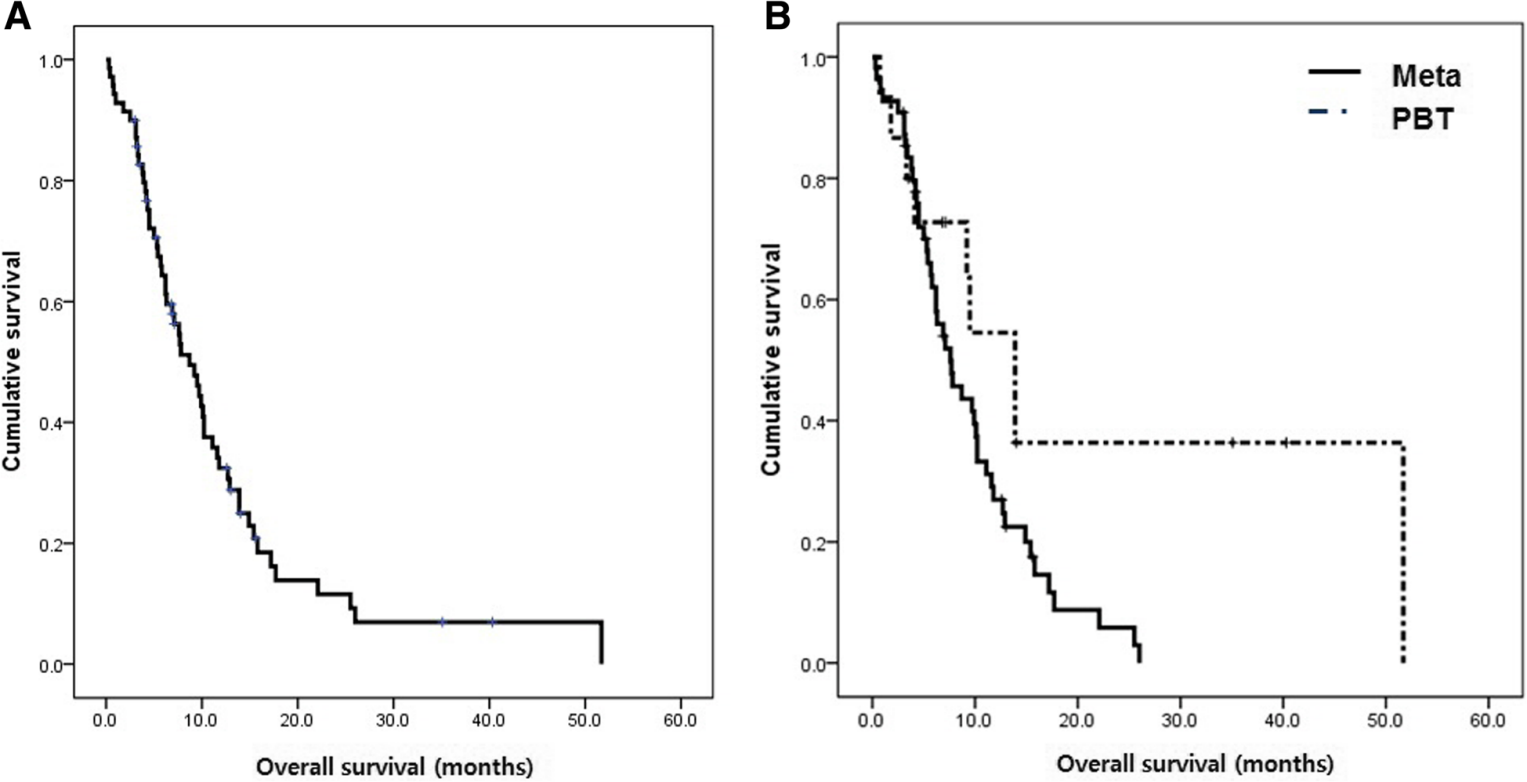
Overall survival (OS) of patients who received CSF shunts due to hydrocephalus from leptomeningeal carcinomatosis (**a**). OS according to preceding disease of brain metastases and primary brain tumors (**b**) (*n* = 70)

Fifty-six patients (80%) died during follow-up, 27 (48%) because of LMC progression and 13 (23%) because of systemic disease progression. The cause of death was undetermined in 16 patients (29%). The median survival of all patients was 8.7 months (range, 0.2–52; 95% confidence interval (CI), 6.0–11.4; Fig. 1) after LMC diagnosis and 3.9 months (95% CI, 2.6–5.2) after initial shunt surgery.

Patients with LMC from systemic cancer had significantly shorter OS than patients with primary brain tumors (7.6 vs. 13.9 months, *p* = 0.03, Fig. 1b). Patients with breast cancer had longer OS than patients with NSCLC (17.2 vs. 7.6 months, *p* = 0.044).

**Fig. 2:**
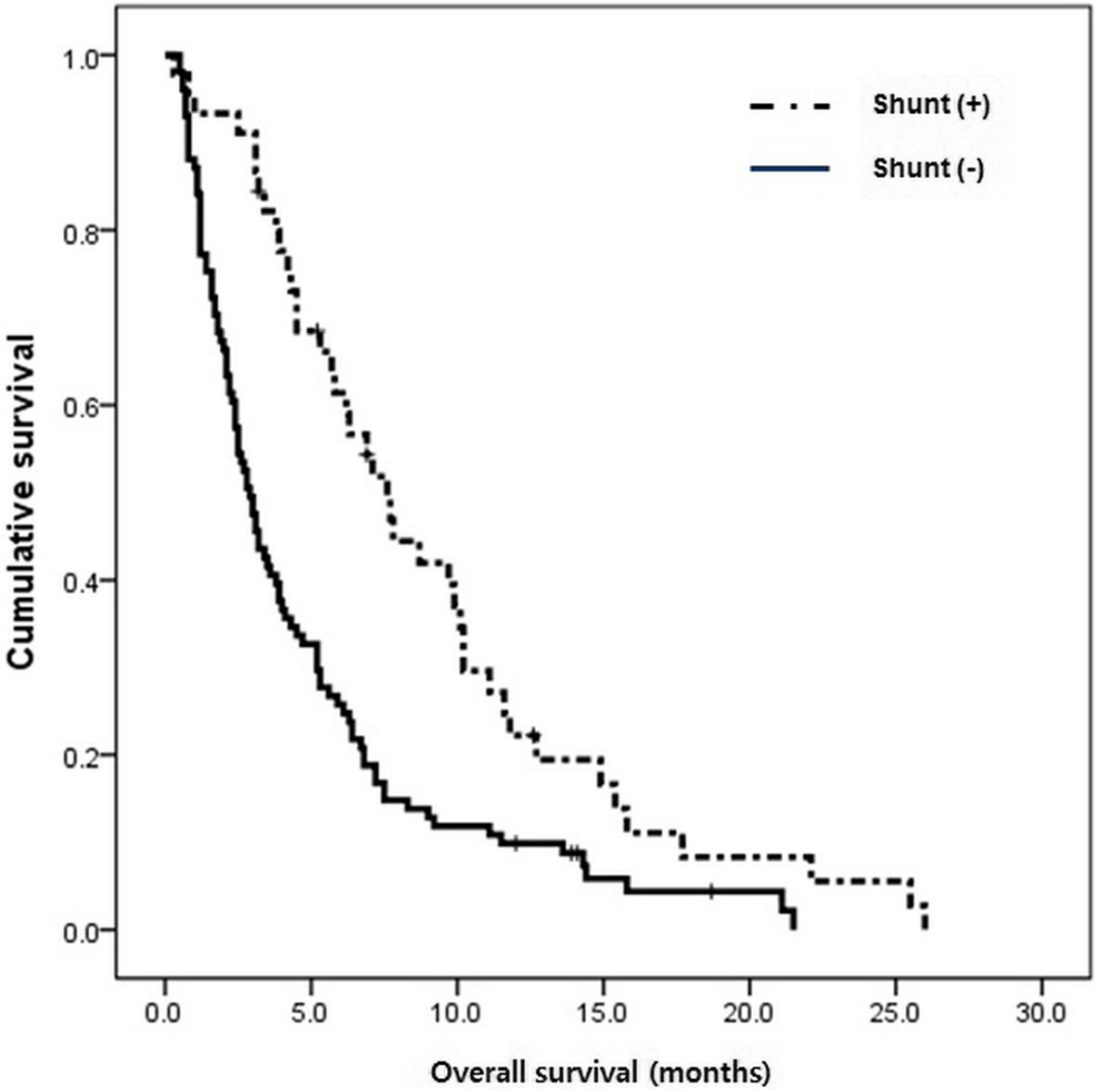
Comparison of overall survival time of patients with non-small cell lung cancer that received shunt surgery (*n =* 45) versus that of patients with non-small cell lung cancer that did not receive shunt surgery but received conventional intraventricular chemotherapy (*n* = 101; data published in J Thorac Oncol, 2013 [17])

The OS of patients with LMC from NSCLC (*n* = 45) was compared to patients from another set of patients (*n* = 46) with LMC from NSCLC who had increased ICP and received intraventricular chemotherapy (*n* = 105) but did not receive shunts in a historical data [17]. The OS of NSCLC patients with the shunt (7.6 months; 95% CI, 5.8–9.4) was significantly prolonged compared to that of NSCLC patients without the shunt (2.3 months; 95% CI, 1.6–3.0) (*p* < 0.001, Fig. 2).

## Discussion

Despite the poor prognosis associated with LMC, CSF shunting to treat hydrocephalus or increased ICP significantly attenuated symptoms and prolonged OS in this study.

### Effectiveness of CSF shunt in patients with LMC

Most studies reporting clinical results of CSF shunts in patients with LMC have focused on the analysis of LMC or CNS metastases, and few patients with those conditions receive shunt procedures [11, 18]. Omuro et al. [19] reviewed the outcomes of 37 patients with LMC from systemic cancer (excluding primary brain tumors) that received VP shunt for increased ICP and reported improvement of ICP-related symptoms in 27 patients (77%). Lee et al. [13] reported a similar improvement rate of 80% after VP shunt in patients with CNS metastases (40 patients with LMC and 10 patients with parenchymal brain metastases), noting improvement of headache (86% of patients), gait disturbance (71% of patients), cognitive dysfunction (40% of patients), and urinary incontinence (40% of patients). In the present study, 59 patients (84%) had improvement of preoperative symptoms, including 24 patients (34%) whose condition was “normalized.” Future studies will need to differentiate between symptoms caused by hydrocephalus and those caused by LMC (e.g., altered mentality) or brain tumors, in case of accompanying parenchymal mass lesions.

Despite the improvements in symptoms, some shunt surgeries resulted in revisions due to malfunction or infection. Complication rates following shunt procedures vary from 4 to 85% according to the shunt type (VP vs. LP), procedure (freehand vs. stereotactic), valve type (programmable vs. fixed), or chronology [20–23]. In a nation-wide (the USA) study of revision rates after shunt surgery in 4480 patients with idiopathic intracranial hypertension from 2005 to 2009, Menger et al. reported that 3.9% of VP shunts and 7.0% of LP shunts (*p* < 0.0001) required revision [15]. The revision rate in patients with LMC appears to be higher than those figures. In this study, LP shunts with fixed pressure-valve reservoirs had the highest revision rate (5 out of 7) among the shunt and reservoir types; VP shunts malfunctioned in three out of 51 patients (5.9%). Omuro et al., who used only VP shunts for patients with LMC, reported that 8% of the shunts malfunctioned and required revision surgery. Neither the present study nor that of Omuro et al. found cancer cell obstruction of the shunt tubing or the reservoir. Another dangerous potential complication of CSF shunts is peritoneal seeding of cancer cells. To date, the documented cases of such seeding are mostly from medulloblastomas, which are well known to cause extra-neural metastases [14, 24, 25]. In those cases, the shunt was inserted before or after tumor removal, and peritoneal seeding occurred with or without primary tumor recurrence. Although the five patients with medulloblastoma in the present study had no documented shunt-mediated metastasis, such metastasis has been diagnosed up to 5 years after shunt placement [25].

### Overall survival after the shunt

VP shunt has been suggested to relieve increased ICP and to improve the OS of patients with LMC [6, 9, 11]. Omuro et al. reported median OS among 37 patients with LMC from systemic cancer as 2 months after the shunt and 4 months after diagnosis of LMC (range, 2 days to 3.6 years) [19]. Jung et al. reported that patients with LMC and hydrocephalus that received surgical treatment had longer OS than those that did not receive surgical treatment (5.7 months vs. 1.7 months), although the difference was not statistically significant because of the small numbers of patients (*n* = 7 and 11, respectively) [11]. In the present study, patients with LMC from systemic cancer showed a median OS of 7.6 months after diagnosis.

### Appropriate shunt types in patients with LMC

Both VP and LP shunting could safely divert CSF flow either from a ventricle or spinal arachnoid space to peritoneal space. Each type of shunt system has advantages and disadvantages, so the choice should be tailored to the patient’s condition. In general, LP shunting is confined to communicating hydrocephalus (HCP) and preferentially used in patients who are not suitable for cranial surgery (i.e., idiopathic intracranial hypertension with slit ventricle) or who want to avoid cranial surgery, whereas VP shunting can be used regardless of communicating or non-communicating HCP [15, 26]. Earlier-era LP shunts without adjustable valve reservoirs had problems in adjusting the amount of CSF drainage and assessing the shunt patency [27, 28]. Hence, LP shunts have been less frequently used than VP shunts. Those problems are much reduced since the advent of LP shunts with programmable valve reservoirs [29]. In the present study, all mechanical malfunctions of LP shunts occurred with the fixed-valve reservoir type; LP shunts with programmable valve reservoirs had revisions due only to infection.

Continuing intraventricular chemotherapy was tried in patients with LMC and a shunt. For those with VP shunt, CSF is directly drained into the peritoneal space from the ventricle, and therefore, a drug delivered intraventricularly would be mainly carried not into lumbar/cisternal CSF space but into the peritoneal space. To overcome this problem, on-off valve system had been tried in other studies for the purpose of enforcing drug delivery to lumbar/cisternal CSF space by closing the shunt [30]. However, we still have not had pharmacokinetic data that showed how much of the intraventricularly injected drug reached lumbar/cisternal CSF space during valve-off time in these patients who lost their physiologic CSF flow unless they injected the drug via lumbar puncture. In this study, we used a combination of Ommaya reservoir and LP shunt. Previously, this concurrent use of Ommaya reservoir and LP shunt was studied for the access to CSF space in the era of LP shunt without reservoir valve system. Zhang et al. reviewed the LP shunting in patients with LMC and suggested a hypothetical advantage of cooperative use of Ommaya reservoirs [31]. Eleven patients in the present study received an LP shunt in addition to a pre-installed Ommaya reservoir for the purpose of intraventricular chemotherapy. We measured lumbar methotrexate level from LP shunt reservoir in a patient and evaluated therapeutic concentration was achieved at approximated half-time of 2.3–4.2 h (data not provided). Four of those patients contracted *S. epidermidis* infections after intraventricular chemotherapy injection. Recent improvements in aseptic techniques of intraventricular chemotherapy injection have so far resulted in no further CSF infections in our institution.

## Conclusion

Patients with LMC and increased ICP or hydrocephalus benefit from CSF shunt in terms of symptom alleviation and survival. Although shunt malfunctions and infections may occur, careful aseptic technique and sophisticated programmable valve systems decrease the procedure-related complications.

## Abbreviations

CSF: Cerebrospinal fluid
HCP: Hydrocephalus
ICP: Intracranial pressure
KPS: Karnofsky performance status
LMC: Leptomeningeal carcinomatosis
LP: Lumboperitoneal
NSCLC: Non-small cell lung cancer
OS: Overall survival
VP: Ventriculoperitoneal
